# Genetic and morphological support for possible sympatric origin of fish from subterranean habitats

**DOI:** 10.1038/s41598-018-20666-w

**Published:** 2018-02-13

**Authors:** Iraj Hashemzadeh Segherloo, Eric Normandeau, Laura Benestan, Clément Rougeux, Guillaume Coté, Jean-Sébastien Moore, NabiAllah Ghaedrahmati, Asghar Abdoli, Louis Bernatchez

**Affiliations:** 1 Department of Fisheries and Environmental Sciences, Faculty of Natural Resources and Earth Sciences, Shahr-e-Kord University, Shahr-e-Kord, P. B. 115 Iran; 2 Département de biologie, Institut de Biologie Intégrative et des Systèmes (IBIS), Pavillon Charles-Eugène-Marchand 1030, Avenue de la Médecine Université Laval, Québec, Québec G1V 0A6 Canada; 3 Lorestan Department of Environment, KhoramAbad, Iran; 4 Department of Biodiversity and Ecosystem Management, Environmental Sciences Research center, Shahid Beheshti University, Tehran, Iran

## Abstract

Two blind Iran cave barbs, *Garra typhlops* and *Garra lorestanensis*, exist in sympatry in a single subterranean habitat, raising the hypothesis that they may represent a case of sympatric speciation following a colonization event. Their different mental disc forms have prompted some authors to propose the alternative hypothesis of two separate colonization events. In this study, we analysed a genome-wide panel of 11,257 SNPs genotyped by means of genotyping-by-sequencing combined with mitochondrial cytochrome c oxidase sub-unit I sequence data, field observations and morphological traits to test these two hypotheses. Field data suggest some degree of ecological divergence despite some possible niche overlap such that hybridization is possible. According to both nuclear and mtDNA data, the cave barb species are monophyletic with close phylogenetic relationships with *Garra gymnothorax* from the Karun-Dez and Karkheh river basins. The historical demography analysis revealed that a model of Isolation-with-Migration (IM) best fitted the data, therefore better supporting a scenario of sympatric origin than that of allopatric isolation followed by secondary contact. Overall, our results offer stronger support to the hypothesis that speciation in the subterranean habitat could have occurred in sympatry following a colonization event from the Karun-Dez-Karkheh basins in the Zagros Mountains of Iran.

## Introduction

Speciation is the keystone process of diversification and can be categorized in geographic and non-geographic modes. There are three recognized geographic modes of speciation: sympatric, parapatric, and allopatric^1–4^. Allopatric speciation is believed to be the most common mode of speciation in nature. Conversely, while generally accepted as a possible outcome, the frequency of occurrence of sympatric speciation in nature is still debated^4–6^. Furthermore, demonstrating the occurrence of sympatric speciation is challenging because species developed in allopatry may come in secondary contact, thereby creating patterns of divergence that can be difficult to distinguish from those that would be expected under sympatric speciation^2^. Therefore, convincing cases of sympatric speciation tend to be restricted to habitats including remote islands, lakes, and caves, where allopatric divergence is unlikely^5,7^. In non-geographic modes of speciation, speciation can be viewed as product of different mechanisms including divergent selection (ecological speciation), genetic drift and hybridization (speciation with no selection), and selection caused by sexual or genetic conflicts (mutation order speciation)^8^.

Some of the best-supported examples of sympatric speciation come from fishes inhabiting geologically young and isolated habitats such as the Crater Lakes in Nicaragua or in African great lakes^5,7,9^. The fishes in these lineages took advantage of ecological opportunities in species-poor habitats to diversify and occupy otherwise empty niches^10^. However, cases of sympatric speciation can be confounded with allopatric speciation^2,5,7^. For instance, in three-spined stickleback (*Gasterosteus aculeatus*), sympatric limnetic and benthic forms within the postglacial lakes in British Columbia developed via two invasion events from their marine progenitor, thus not conforming to sympatric speciation strictly speaking^3,5^. As noted in the case of the Nicaraguan cichlids^7^, the habitats with higher probability for sympatric speciation are the ones with the minimal or no connection to other habitats, making repeated gene flow from other habitats unlikely.

Subterranean habitats, with limited connections to surface habitats, can therefore also be considered suitable contexts for sympatric diversification and speciation. Habitat isolation caused by ecological forces may be observed in the resource-limited subterranean habitats where different fish species coexist. Subterranean habitats are environmentally stable (*e.g*., stable temperature, darkness), but limited in trophic resources and primary production due to low light intensity resulting in a low rate of photosynthesis^11–13^. This light limitation, jointly with an eco-hydraulic zonation of subterranean habitats^11^, makes them an excellent natural context to study the evolution of biodiversity^13^. These features of the subterranean habitats increase the intensity of competition for limited resources and can lead to species/population divergence to maximize the use of variable resources and habitat types while minimizing competition^5,12^. This diversification in habitat use can drive reproductive isolation and ultimately sympatric speciation. Here, we document a possible case of sympatric speciation in a subterranean habitat with two closely related sympatric cyprinid fish species, showing genetic and morphological divergence.

The blind Iran cave barb, *Garra typhlops*, is a labeonin cyprinid fish species known from a single subterranean, well-like habitat in the Zagros Mountains of Iran^14^. Two sympatric forms of the species, one bearing a mental disc and one with no mental disc, occupy the same habitat^15^. Hashemzadeh Segherloo, *et al*.^14^ and Farashi, *et al*.^16^ showed that these two forms are diverged from one another based on mitochondrial *COI* and *cyt-*b sequence data. Recently, the disc-bearing form of the blind cave barb was described as *Garra lorestanensis*^17^. The level of *COI* sequence divergence between *G. typhlops* and *G. lorestanensis* is higher (3.6% Kimura 2-parameter distance (K2P)) than the mean mitochondrial genome divergence values reported at the intra-specific level for marine (0.39% K2P) and freshwater fishes (0.27% K2P)^18,19^. The Cave barb species belong to the *Garra rufa* clade of the Middle Eastern labeonins and are phylogenetically closest to *Garra gymnothorax* that inhabit Karun, Dez, and Karkheh River Basins, among which the Dez River is closest to the Cave Barb locality (5 km apart from the cave barb locality)^20^. Although little is known about their biology, the two species seem to have different habitat preferences. Field surveys suggested that the non-disc-bearing form, *G. typhlops*, is usually present in the stagnant part of the habitat all year round, whereas the disc-bearing form, *G. lorestanensis*, was found to mostly occupy this part of the habitat during the pluvial period (March-May) when water from the cave outflows. In addition, we have recently observed an individual exhibiting an intermediate disc form in the cave barb locality that may be a hybrid, an extreme case of intra-specific variation, or a genetically differentiated species.

According to previous findings and observations, two possible scenarios can be proposed to explain the history and mechanism of speciation in the subterranean habitat. The first scenario involves two invasions, whereby *G. typhlops* invaded the habitat first and diverged from the incipient surface-dwelling species while the colonisation by *G. lorestanensis* occurred later, since *G. lorestanensis* has retained mental disc. In this scenario, the colonization by the second species is followed by character displacement that has shaped the modern cave barb species pair, similar to the case of the stickleback species pairs^3^. This scenario implies that *G. typhlops* would have undergone character displacement towards the use of a distinct trophic niche and to reduce competition with the new coloniser (*G. lorestanensis*). The second scenario would imply speciation in sympatry, whereby only one colonisation event from the source ancestral population was followed by sympatric divergence. In some cases, even with multiple colonisation events the scenario can be considered as sympatric divergence/speciation. Thus, after multiple colonisations from different resources a hybrid swarm may form and then new lineages/species may diverge from the hybrid swarm sympatrically^21^. Unfortunately, there are no fossil records of the genus *Garra*^20^ or data on the geological history of the habitat to be used in reaching inferences on the timing of the colonisation/s or speciation.

Previous genetic studies of blind Iran cave barbs were based on mitochondrial data only^14,16,20^, but the mitochondrial genome represents only a small percentage of the genome and is of maternal origin, which may lead to biases when drawing systematic and taxonomic conclusions. Indeed, mitochondrial data may be affected by events such as incomplete lineage sorting, introgression or selection, which may disrupt the phylogenetic signal^22–24^. Moreover, uniparental genetic data are not sufficiently informative to make inferences on the biological relationships of sympatric or syntopic species^25^. Next generation sequencing (NGS) technology provides the possibility of analysing thousands of genome-wide bi-parental markers for multiple individuals^26–29^. This approach can be efficiently applied to both model and non-model species for which no genome data is available^26,27,30^. In addition, the development of efficient software packages, including the STACKS pipeline^27^, has provided a suitable set of tools for handling the large amounts of data produced by NGS techniques^31^. More particularly in hybridized populations, genomic techniques may be useful for the identification of multiple species-diagnostic markers that would allow precise estimates of population and individual-level admixture^32^.

The main goal of this study was to shed light on the possible mechanism of cave barb speciation (whether sympatric or allopatric) regarding the scenarios described above. In this regard, we consider the criteria proposed to differentiate cases of sympatric speciation from those of allopatric speciation^4^. According the criteria proposed by Coyne and Orr^4^, speciation would be sympatric if the species: (a) have large or complete geographic overlap, (b) are completely diverged, (c) should be monophyletic or sister species, and (d) are unlikely to have evolved in allopatry during their evolutionary history. We analyse these criteria for the cave barb speciation using temporal field data, genomic analysis (using the GBS method), mitochondrial sequences (*COI*), and morphological comparisons.

## Results

### Morphological analysis

**Figure 1:**
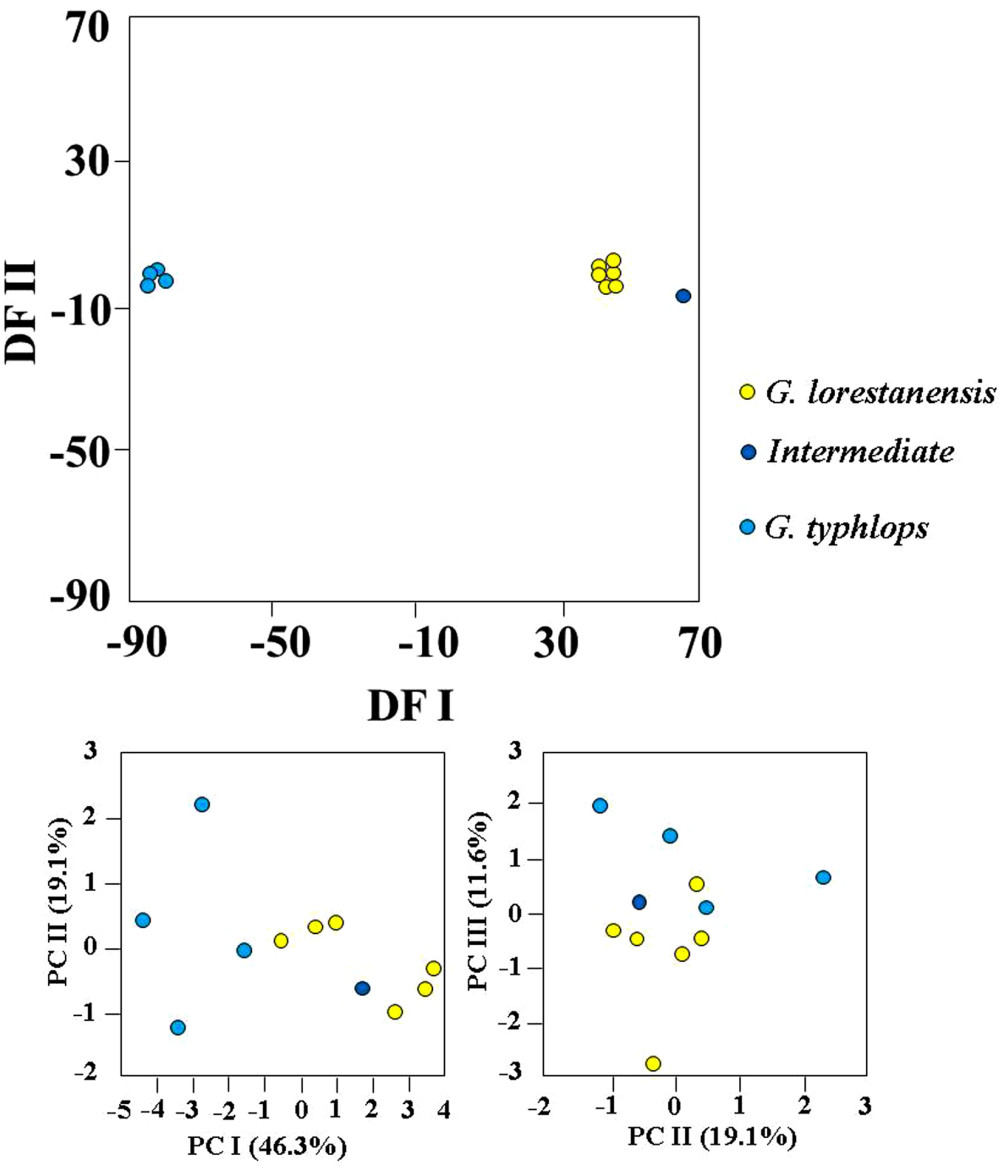
Top panel: Discriminant function (DF) analysis for the morphometric variables of the cave barb forms; Bottom panel: principal component analysis (PCA) of the cave barb morphometric variables. The 1^st^, 2^nd^, and 3^rd^ components are graphed against each other.

**Figure 2:**
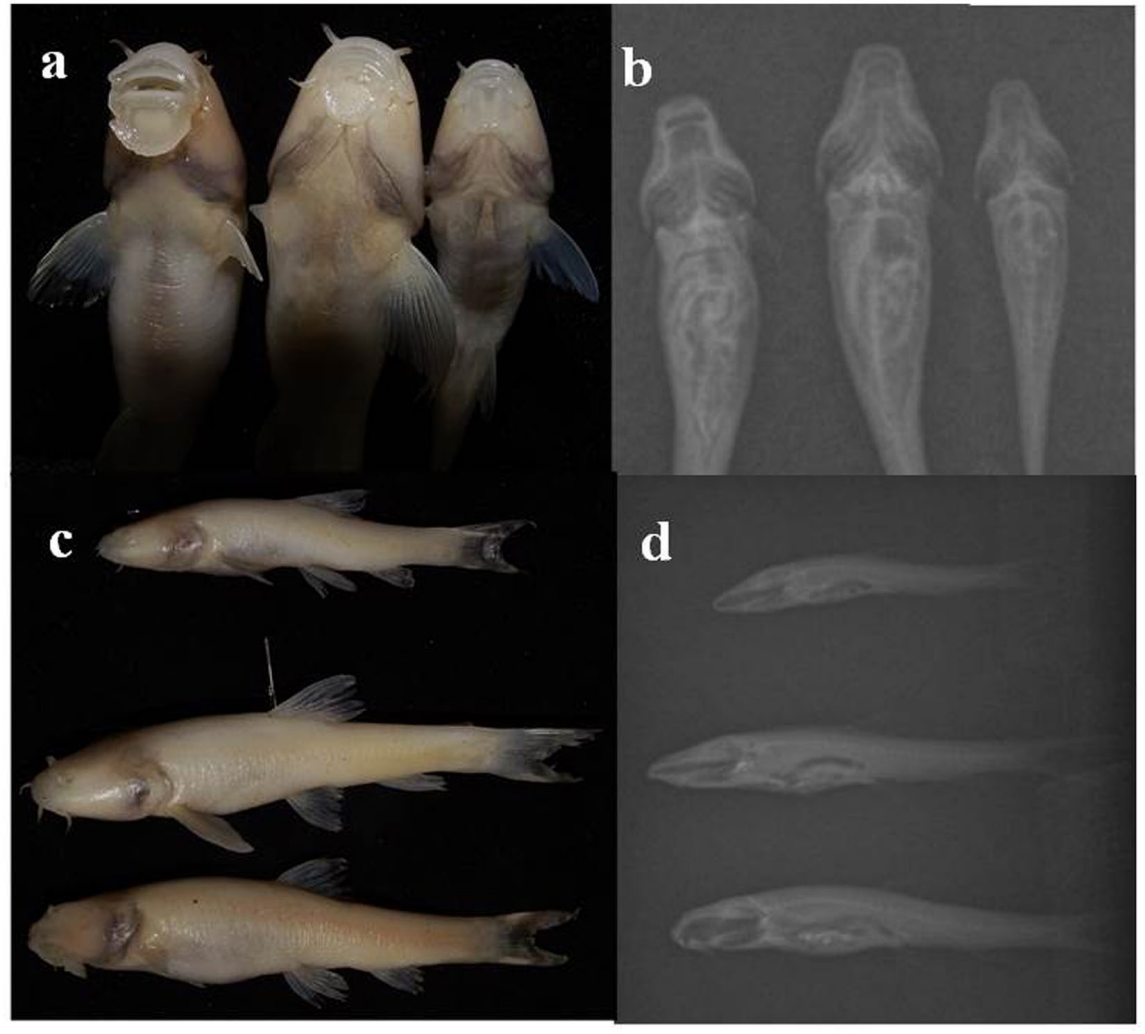
The different sympatric mental disc forms in blind Iran cave barb; (**a**): from right to left: well-developed disc, intermediate disc, and reduced disc, and (**b**): the corresponding ventral x-ray view of different disc forms; (**c**): from up to bottom: reduced disc, intermediate disc, and fully developed disc, and (**d**): the corresponding x-ray side view of different disc forms.

Discriminant function analysis of the morphological variables produced two discriminant functions plotted against each other (Fig. 1). The *G. typhlops* and *G. lorestanensis* groups were completely separated on the discriminant function plot (Wilks Lambda = 0.000; Approx. F = 71.897; *P* < 0.001). The intermediate form positioned closer to the disc-bearing species, *i.e*., *G. lorestanensis*. The most important morphological variables in discriminating the groups were L8–10 (origin of the anal fin to the base of the pectoral fin; *F*-to-inter = 1017.15; Wilks Lambda = 0.0346; Approx. F = 15.314; P < 0.001), L2-9 (origin of dorsal fin to the base of the pelvic fin; *F*-to-inter = 94.88; Wilks Lambda = 0.0005; Approx. F = 33.384; P < 0.001), L2-6 (origin of dorsal fin to the lower end of the caudal peduncle; *F*-to-inter = 61.17; Wilks Lambda = 0.0979; Approx. F = 36.862; P < 0.001), L6-9 (lower end of the caudal peduncle to the base of the pelvic fin; *F*-to-inter = 43.55; Wilks Lambda = 0.0055; Approx. F = 15.554; P < 0.001), and L4-9 (upper end of the caudal peduncle to the base of pelvic fin; *F*-to-inter = 15.73; Wilks Lambda = 0.0000; Approx. F = 71.901; P < 0.001). The positioning of the mouth opening is nearly terminal in *G. thyphlops* and the intermediate form compared to *G. lorestanensis* (Fig. 2a and b). In assignment tests using a jack-knifing method based on the discriminant functions and the best discriminating variables noted above, all individuals were correctly assigned to their original groups. The overall correct assignment rate was 100%. On PCA plots, the species were not separated completely (Fig. 1).

### Mitochondrial phylogeny

**Figure 3:**
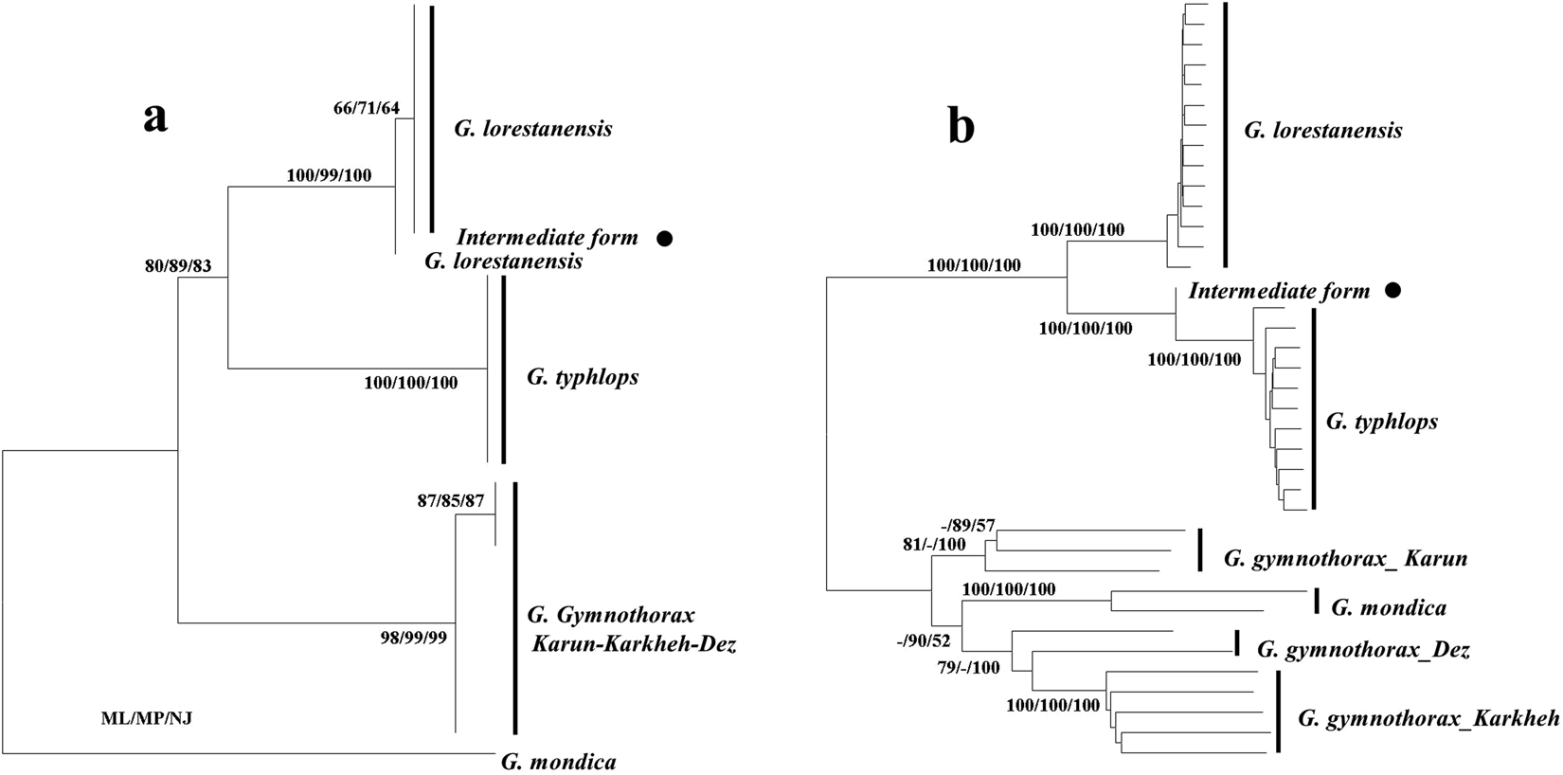
(**a**) Maximum Likelihood phylogram reconstructed for a 558-bp 5′ *COI* sequence. The values on the branches are bootstrap support values for Maximum Likelihood, Neighbour-Joining, and Maximum Parsimony methods, respectively. (**b**) Maximum Likelihood phylogram reconstructed for a concatenated 563,840-bp nucDNA sequence produced by next generation sequencing. The values beside the branches are maximum likelihood (ML), neighbour-joining (NJ), and maximum parsimony (MP) boot-strap support values, respectively. The filled circles on both trees denote the intermediate disc-bearing form of cave barb.

The best model for *COI* data based on the AIC information criterion was TrN (Tamura-Nei) + Gamma. The phylograms reconstructed using Maximum Likelihood, Neighbour-Joining, and Maximum Parsimony approaches were all similar in their topology, and all groups analysed were supported with moderate to high boot-strap values (Fig. 3a). On the phylograms, *G. lorestanensis, G. typhlops*, the intermediate cave barb individual (nested in a sub-clade as sister groups: BS = 80–89), and *G. gymnothorax* from the Karun and Karkheh basins formed distinct and highly supported monophyletic clades (BS = 98–99). The intermediate form nested within the *G. lorestanensis* sub-clade (Fig. 3a). The mean between-group K2P distances were highest between *G. typhlops* and *G. mondica* (6.7%) and *G. lorestanensis* and *G. mondica* (6.3%). The least mean between-group K2P sequence distance was calculated for *G. typholps* and *G. lorestanensis* (3.6%). The K2P distances between *G. gymnothorax* from Karun-Dez basin and *G. typhlops* and *G. lorestanensis* were 4.6 and 4.3%, respectively.

### Nuclear DNA (nucDNA) phylogeny

**Figure 4:**
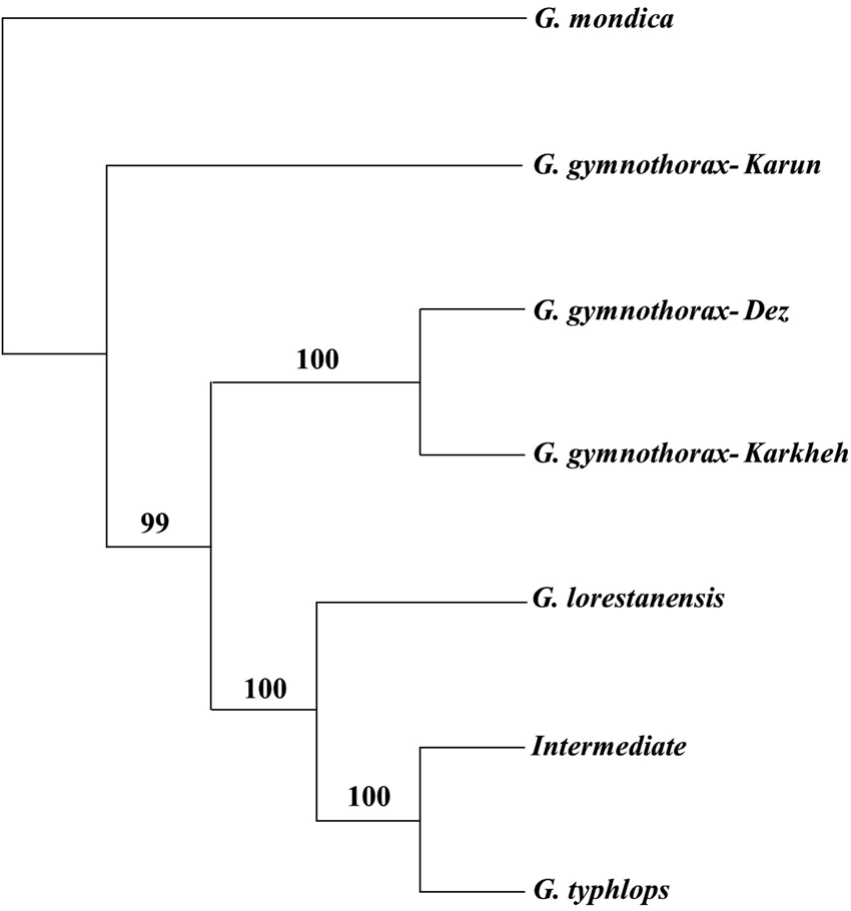
Fifty present majority rule consensus Species tree reconstructed for SNP loci using SVDquartets method. The values on the branches are bootstrap support values calculated implementing 1000 permutations.

A total of 11,257 filtered SNPs were identified among the 38 analyzed specimens. Different evolutionary models produced similar results, and thus we did not detect any model sensitivity in genomic data. The phylograms reconstructed using the model-based (ML) and non-model based approaches were similar in topology and, hence, only one of the phylograms is presented with the boot-strap support values for all methods noted (Fig. 3b). On the phylogram reconstructed using genomic sequence information, *G. typhlops* and *G. lorestanensis* nested in a well-supported monophyletic clade composed of two sub-clades each pertaining to one of the disc forms with robust bootstrap supports (BS = 100). In contrast to the mtDNA results, the intermediate disc-bearing specimen (Fig. 3b) nested within the *G. typhlops* sub-clade. *Garra gymnothorax* genomic sequences nested in two highly supported clades, one including the specimens from Karun and one including the specimens from Dez and Karkheh basins. On the species tree reconstructed using SNP data also a pattern similar to what observed on Genomic and mtDNA gene trees was observed: both cave barb species were monophyletic with maximal bootstrap support (Fig. 4). The Da differentiation between the cave barb species was 0.13%, while the respective distances of the cave barb species relative to other none cave *Garra* species considered here varied between 0.25% and 0.32%.

### Admixture and clustering analysis

**Figure 5:**
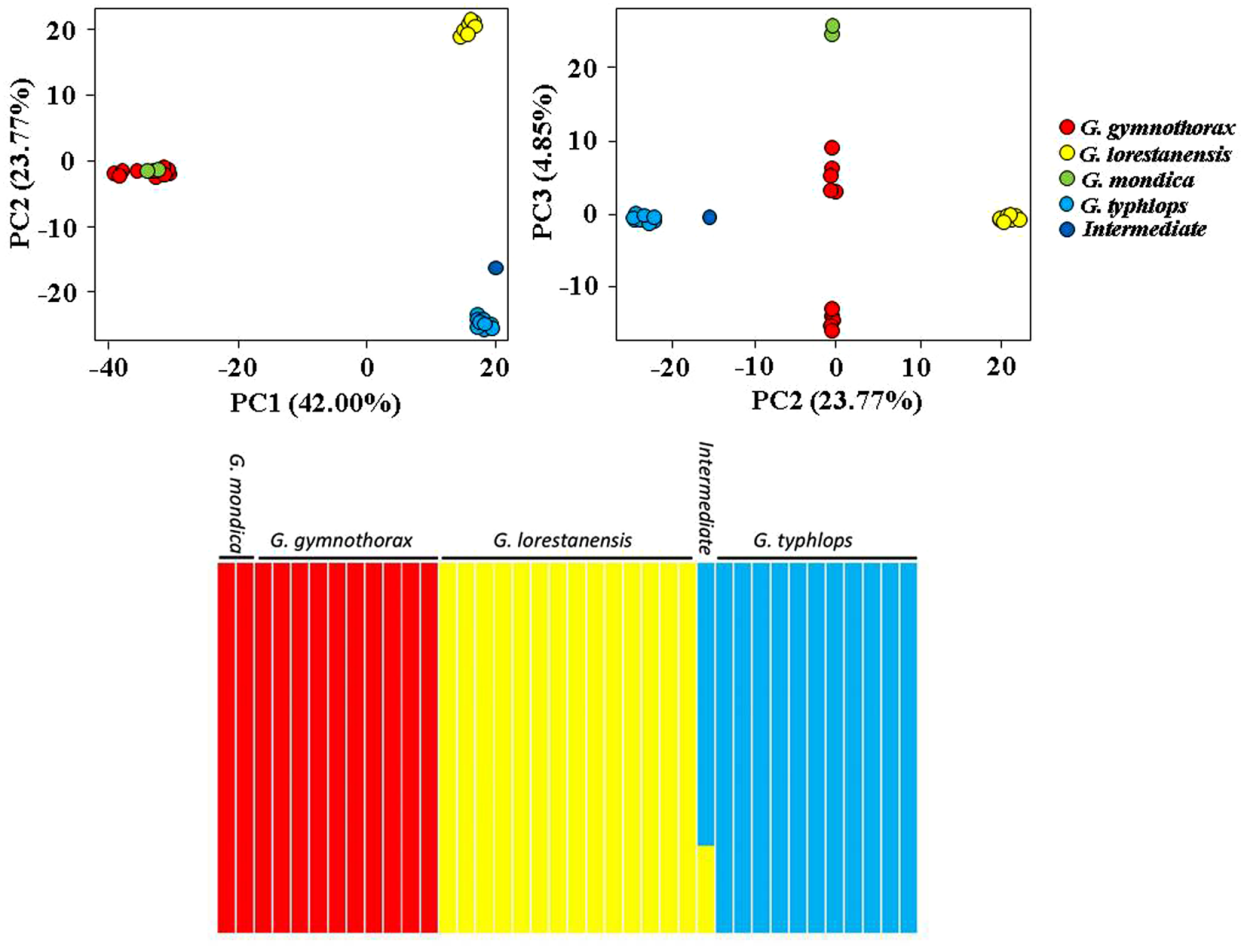
Top panel: Principal component analysis of SNP variance between the cave barb species and the surface dwelling out-group species. Bottom panel: Bayesian clustering pattern from a STRUCTURE analysis for K = 3 (Clustering pattern was similar for K = 3–5).

Using all 11,257 SNPs, the STRUCTURE analysis was consistent with the existence of three distinct genetic groups corresponding to the different species. Structure results showed that the intermediate specimen shared more ancestry (77%) with *G. typhlops* than with the disc-bearing form (23%), *G. lorestanensis* (Fig. 5). Principal Components Analysis (PCA) of SNP data revealed that the cave barb species are diverged from one another and from the out-group species used here. The cave barb species show divergence on the second principle component (PCII) but they are diverged from the out-groups on both PCs (Fig. 5). The PCA showed that the morphologically intermediate individual also falls genetically intermediate between the two cave species but closer to *G. typhlops* (Fig. 5).

### Mode of divergence

**Figure 6:**
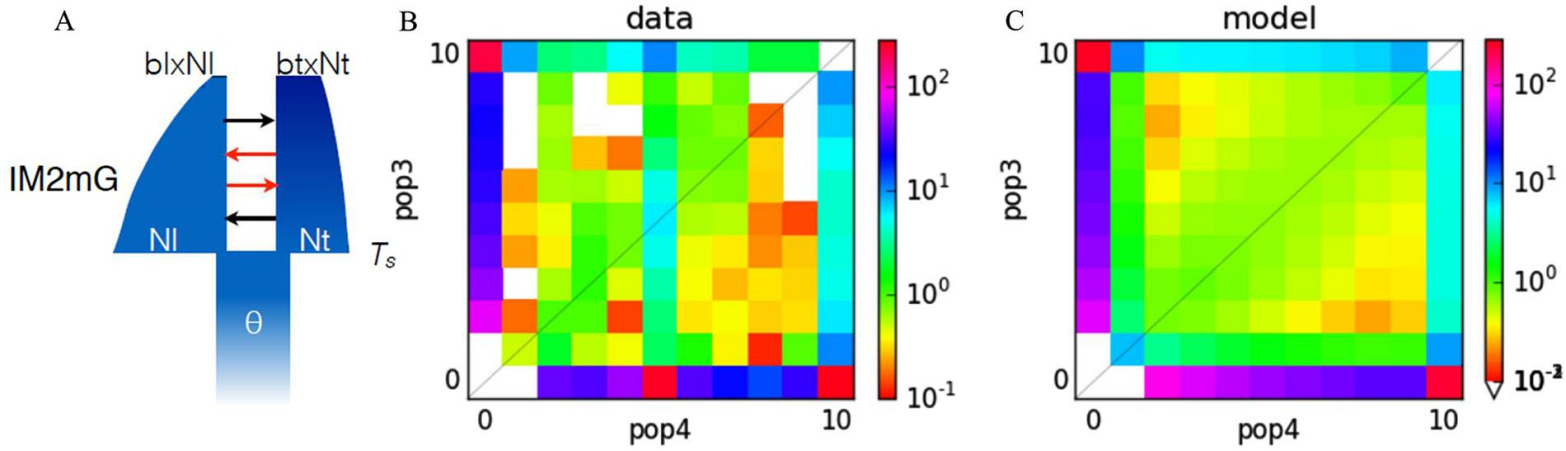
Historical demography of the species-pair: (**A**) IM2mG scenario corresponding to the best-fitting model to the empirical Joint Allele Frequency Spectrum (JAFS). (**B**) Observed JAFS for *G. lorestanensis* (*y*-axis) and *G. typhlops* (*x*-axis) populations and (**C**) the predicted JAFS of the best-fitting model. The color scale corresponds to the probability of occurrence of the derived allele from five individuals from both populations.

The inferred *O* parameter gave us the proportion of well-oriented markers for each model, reflecting good quality of the markers used for this analysis. While the JAFS (Joint Allele Frequency Spectrum) dimensions were reduced, only two models among the 14 tested outperformed others based on the ∆*AIC* and w*AIC*. We retained the IM2mG and AM2mG models with weights of 0.96 and 0.04, respectively. Considering only the best model with the higher wA*IC* value, the best-fitted model to the JAFS was IM2mG (Fig. 6 & Fig. SI), suggesting a sympatric mode of speciation with heterogeneous gene flow between diverging species that experienced bottlenecks during the divergence. The heterogeneous gene flow indicated that only a portion of loci were under disruptive selection and putatively involved in reproductive isolation. The inferred effective population sizes showed an asymmetric pattern with a higher *N*_e_ for *G. lorestanensis* and asymmetric gene flow between the two populations (Table 1).

**Table 1:** Model-fitting results.

MODEL	*K*	MLE	*AIC*	*∆*_*AIC*_	*w*_*AIC*_	*θ*	*N*_*l*_	*N*_*t*_	*b*_*l*_	*b*_*t*_	*m*_*et-*>*l*_	*m*_*el-*>*t*_	*m*_*e*′t*-*>*l*_	*m*_*e*′*l-*>*t*_	*Tsplit*	*Tpost-split*	*P*	*O*
IM2mG	11	−489.62	1001.25	0	0.96	471.591	3.402	2.375	0.052	0.103	0.000	35.06	0.007	0.000	—	1.136	0.064	0.988
AM2mG	12	−491.86	1007.72	6.47	0.04	408.763	1.448	3.128	0.160	0.082	0.043	4.679	1.872	0.097	0.368	1.24	0.875	0.988
SIG	6	−499.64	1011.28	10.04	—	536.866	2.529	3.799	0.055	0.036	—	—	—	—	—	0.776	—	0.99
AMG	9	−499.80	1017.60	16.35	—	634.21	0.987	1.778	0.107	0.062	2.714	0.017	—	—	0.079	0.451	—	0.989
IMG	8	−503.20	1022.41	21.16	—	429.283	1.299	13.844	0.170	0.013	0.161	0.007	—	—	—	1.398	—	0.979
IM2m	9	−510.44	1038.88	37.63	—	686.362	0.206	0.254	—	—	70.425	0.006	0.003	0.003	—	0.461	0.053	0.989
SC2m	10	−512.04	1044.07	42.82	—	674.396	0.227	0.286	—	—	4.179	148.63	0.039	0.002	0.103	0.418	0.083	0.989
SC2mG	12	−511.50	1047.00	45.75	—	594.828	0.306	0.343	0.111	0.639	0	0	0	0.042	0.472	0.052	0.254	0.99
AM2m	10	−515.21	1050.41	49.17	—	550.991	0.305	0.417	—	—	33.119	0.389	0.033	0.000	0.854	0	0.079	0.987
SI	4	−521.51	1051.02	49.77	—	681.26	0.194	0.242	—	—	—	—	—	—	—	0.395	—	0.989
IM	6	−521.24	1054.48	53.23	—	718.512	0.166	0.207	—	—	0.011	0.001	—	—	—	0.336	—	0.988
SC	7	−520.91	1055.81	54.57	—	703.699	0.177	0.220	—	—	0.000	0.000	—	—	0.357	0.004	—	0.989
AM	7	−521.70	1057.39	56.15	—	713.546	0.166	0.213	—	—	0.001	0.000	—	—	0.133	0.194	—	0.989
SCG	9	−524.17	1066.34	65.09	—	490.12	0.421	2.071	0.106	0.000	0.000	0.000	—	—	0.879	0.000	—	0.988

Parameter values for all 14 models tested for cave barb species. The parameters include maximum likelihood (MLE) related to the model with the least Akaike Information criterion (*AIC*), ∆_*AIC*_ value for each model, weighted *AIC* value (*w*_*AIC*_), the scaling factor (*θ*), the population sizes during different generations *(N*_*l*_ and *N*_*t*_*)*, expansion or bottleneck events *(b*_*l*_*, b*_*t*_*)*, migration rates occurring with a fixed proportion from *G. typhlops to G. lorestanensis* and vice versa *(m*_*et-*>*l*_*, m*_*el-*>*t*_*)*,migration rates with different rates from *G. typhlops to G. lorestanensis* and *vice versa (m*_*e*′*t-*>*l*_*, m*_*e*′*l-*>*t*_*)*,initiation time of divergense (*T*_*split*_), time after divergence initiation (*T*_*post-split*_), the proportion of the genome which evolves neutrally (*P*), and the proportion of the SNPs with correct orientation (*O*).

## Discussion

The three geographic modes of speciation, *i.e*., allopatry, parapatry and sympatry, differ in geographic means of isolation and in the rate of gene flow among the diverging populations^2,4,5^. Allopatric speciation necessitates the complete geographic and therefore reproductive isolation of the populations, and most cases of speciation are categorized in this class^2,4,33^. On the other hand, parapatric and sympatric modes of speciation occur when reproductive isolation and speciation occur while distribution overlaps and there are variable rates of gene flow among populations^2^. To determine whether sympatric divergence occurred and led to speciation four criteria must be fulfilled: (a) sympatric distribution, (b) genetic evidence for reproductive isolation, (c) monophyly, and (d) unlikeliness of allopatric differentiation^2,4,5,7^. Below we interpret and discuss the results obtained for the blind Iran cave barb species according to these criteria.

### Mode of Speciation and species origin

The blind Iran Cave barb species, *G. typhlops* and *G. lorestanensis*, exist in sympatry in the subterranean habitat. Their probably confined nature of the subterranean habitat makes this system a plausible case for sympatric speciation. The only known connection of the subterranean habitat with the surface habitat is through a small stream (known as Kayeh-ru) created during pluvial periods that drains from the cave barb locality to Sirum Stream and then into the Sezar River. The Kayeh-ru stream passes two high waterfalls (7–8 m) that block the upstream migration of *Garra* species from the river to the subterranean habitat (cave barb locality). Bayesian clustering analysis (structure plot) of the SNP data for different K values (K = 3–5) also shows no ancestry from the surface dwelling specimens (out-group species). Therefore, recent colonization of the cave barb species or ongoing gene flow from the river seems unlikely. In addition, the mtDNA data show that specimens from Dez River (the most proximate river to the cave barb locality), Karun River (the same basin), and Karkheh River (the basin west to the cave barb locality) basins differ from the blind cave barbs, which is concordant with a previous report in which cave barbs were considered^20^. This divergence from the surface-dwelling *G. gymnothorax* inhabiting the Karun-Dez and Karkheh basins may indicate that cave barb lineages have been in isolation from the surface habitats for a prolonged period (see below) or that their closely related surface dwelling populations/species have not been sampled or gone extinct during old drought periods^6^.

The mtDNA and nucDNA phylogenetic analyses show that the cave barb species are phylogenetically diverged, which may imply reproductive isolation of the two cave barb species from one another. However, reproductive isolation appears incomplete, given that the intermediate disc-bearing individual is genetically intermediate between both species, which is not unexpected in cases of incipient sympatric speciation^4–6^, especially in cyprinids^34^. The intermediate individual is probably a post-F_1_ hybrid of *G. lorestanensis* and *G. typhlops*, as it shows 23% of genetic ancestry from *G. lorestanensis* and 77% of its genetic composition from *G. typhlops*. Consequently, the intermediate individual clustered with *G. typhlops* as a sister group on the genomic phylogeny. Although admittedly based on the analysis of one available specimen only, this proves that hybrids between these two species can be fertile and reproductively compatible with pure-species individuals or other hybrids. The mechanism for partial reproductive isolation of the cave barb species is not well understood. They appear to show temporal habitat isolation at the accessible part of the subterranean habitat, with *G. typhlops* being present all year round in the slow-flowing/stagnant part of the habitat and *G. lorestanensis* being present mostly during pluvial periods (March–May) when there is an increase in flow-rate. *Garra* species with reduced or no disc usually are observed in slow-moving or stagnant water bodies and the species with a fully developed disc are mostly observed in fast-flowing watercourses^20^. The mental disc is believed to be a morphological character that evolved in labeonin fishes for maintaining position in fast-flowing habitats^35^. Thus, given also the different habitat zones/partitions in the subterranean biome and their poor productivity^11,13^, it is plausible that the cave barb species may occupy different microhabitats based on the flow rate, perhaps to reduce competition and to utilize different resource environments^36^. In turn, such habitat isolation eventually could lead to the observed partial reproductive isolation and hence indirectly to assortative mating by increasing the chance that members of each species would mate more frequently with conspecific individuals^5^. Nonetheless, their contacts and probable syntopy during the pluvial period (March–May), which falls within the spawning season of most cyprinid fish species in the area^37^, can increase the chance of hybridization and gene flow between the two species, as revealed also by the JAFS analysis. One other mechanism opposing gene flow between the cave barbs can be inferred as formation of species barriers which had been reported to start at Da divergence levels as low as 0.075% (Da between cave barbs = 0.13%) between semi-isolated species^38^. Admitedly, a rigorous test of these hypothetical scenarios of flow-dependent habitat isolation or formation of species barriers will require a more extensive study with increased sample size for both pure and hybrid specimens encompassing other parts of the subterranean habitat. Given the scarcity of these taxa, this may however prove challenging. Both phylogenies and morphological data confirm the taxonomic status of the cave barb species reported previously^14–17,20^. On both the mitochondrial and genomic phylogenies, cave barb species nest as sister groups. The mtDNA phylogeny shows deeper genetic divergence between the two cave barb species (3.6% K2P) compared to the genomic divergence (0.14% K2P). The deeper mtDNA in face of a shallow nuclear genomic divergence between the cave barbs can be justified by the 5–10 times higher mutation rate and smaller effective population sizes of mtDNA compared to nucDNA^25,39^. The higher mutation rate and the lower effective population size of mtDNA in combination with behavioural and ecological characters like differential habitat dependence of male and female cave barbs, in which females show philopatry while males disperse more^25,40^, may magnify the divergence depth difference observed between the maternally inherited genome and the nuclear genome. Other possibilities to this differential divergence may be the mito-genomic interactions and selection^40^. Anyway all these possibilities await more trough analyses on behavioural differences, the effects of selection, and mito-genomic interactions.

The historical demography inferred from the Joint Allele Frequency Spectrum (JAFS) revealed that a model of Isolation-with-Migration (IM) with ongoing asymmetrical gene flow best fitted the data, which may be predicted based on Da value of 0.13% between the cave barb species. Roux *et al*.^38^ infer that in species pairs with Da values lower than 0.5% ongoing gene flow is highly supported. Nevertheless, while likely more recent than the divergence from *G. mondica* and *G. gymnothorax*, the mitochondrial divergence of 3.6% is suggestive of a fairly ancient divergence of the two blind cave barb species. Although any molecular clock must be used and interpreted very cautiously, rates reported for cyprinid fish mtDNA cyt-b varies from 0.52%^41^ to 0.76%^42^ per million years, suggesting a divergence time of about 5–6 million years.

Overall, according to demographic history modeling, PCA analysis, habitat overlap, and phylogenetic relationships, the most parsimonious scenario for the origin and current sympatric occurrence of *G. lorestanensis* and *G. typhlops* is likely sympatric speciation with still-incomplete reproductive isolation. As the subterranean habitat is confined and the only known connection to the closest riverine habitat in the Dez basin is through the Kayeh-ru stream, it seems most probable that the ancestral taxon originated from the Sezar River located nearly 5 km away from the cave barb locality (Dez basin) and subsequently to have diverged in sympatry in the subterranean habitat. Admitedly, we cannot rule out the possibility that the contemporary surface dwelling populations or species we have collected may not be the actual ancestral species/population of the cave barbs. To verify that speciation of the cave barbs is indeed a product of one colonization or more, it is reasonable to assume that in the case of a single colonisation event, both species should show (nearly) equal genetic distances relative to the out-group(s), indeed, both the cave barbs are nearly equally diverged from the out-groups (*G. gymnothorax*) in the Karun-Dez basin, with the mtDNA sequence distances being 4.3% and 4.6%, respectively, therefore supporting this view. This was also further supported by the PCA results. While our results are clearly more supportive of a scenario of sympatric origin than that of allopatric origin followed by secondary contact, they are not sufficient to totally rule out other hypotheses including a single or more colonization waves or other mechanisms of speciation. Nevertheless, our results clearly show that this system of sympatric Iran cave barb species deserve further studies pertaining to speciation research.

## Materials and Methods

### Sampling

**Figure 7:**
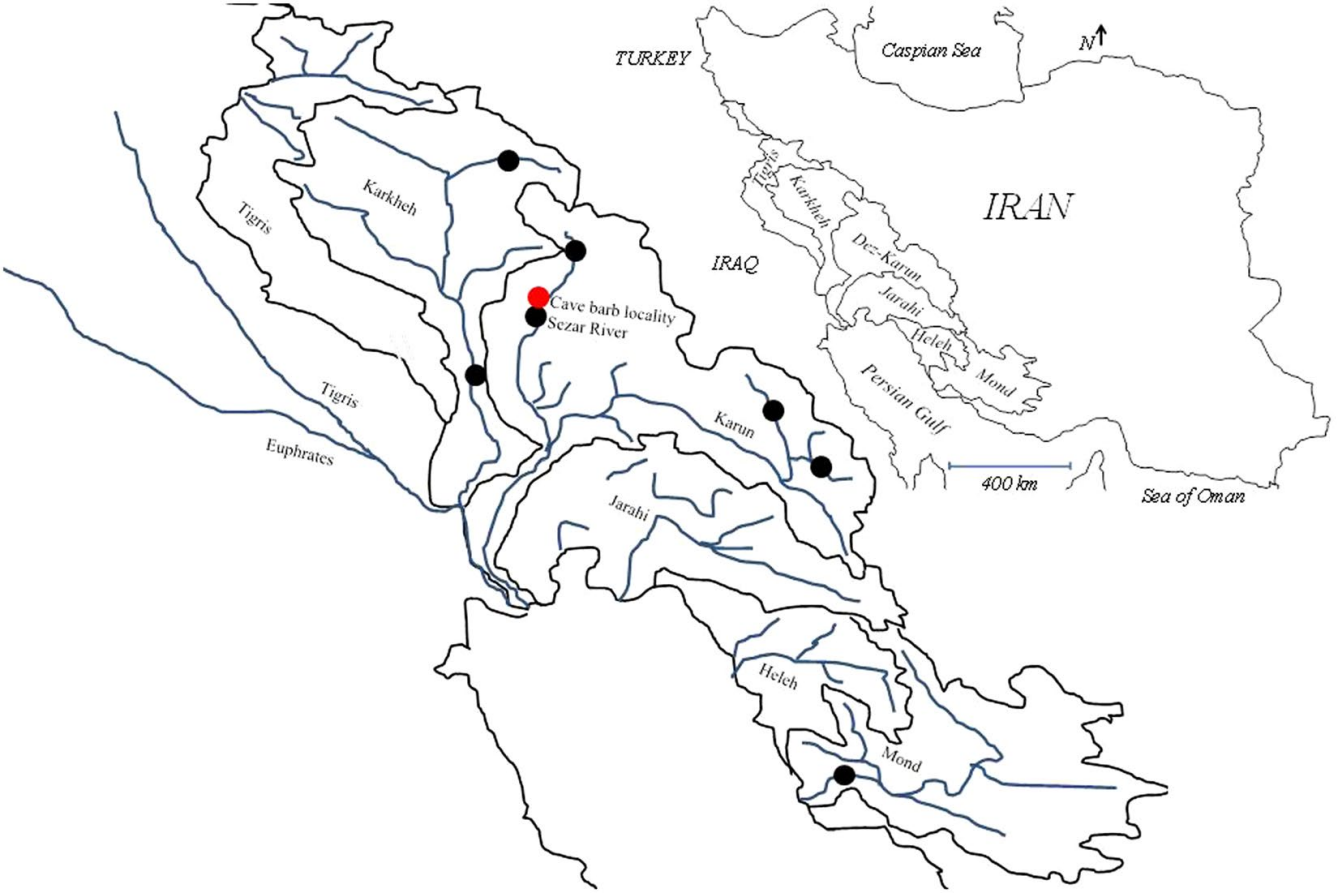
Geographic position of the drainage basins that were sampled. The black circles denote the localities for *G. gymnothorax* and *G. mondica*, and the red circle denotes the cave barb locality. The map was produced using DIVA-GIS v 7.5 and the free spatial data (shape files) available at www.diva-gis.org.

Sampling and collections of the environmental data were conducted from March 2013 to August 2016 in the Zagros Mountains (33°04′38″N 48°35′35″E) using a scope net (Fig. 7). We collected a total of 26 specimens (11 *G. typhlops*, 14 *G. lorestanensis*). In addition, we used 10 *G. gymnothorax* (Karun-Dez and Karkheh basins), and two *Garra mondica* (Mond Basin) specimens for the genomic analysis. We also recorded the frequency of the two species at different times during 2014–2015. Fish were over-anaesthesed using clove powder and the pectoral fin was clipped and preserved in 95% ethanol. The whole fish was preserved in 10% formalin solution except for five fish that were preserved in ethanol. The methods and procedures used during sampling and handling the live fish were all approved by the research and education council of the faculty of Natural Resources and Earth Sciences (Shahr-e-Kord University, Iran) and all are in accordance with the protocols required by the Iranian Department of Environment. The number of fish was restricted according to the permit issued by the Iranian Department of Environment (Permit no. 8613/94). Fish were checked and scored for the presence or absence of mental disc.

### Morphological analysis

Fish specimens were mounted on a Styrofoam board using colored pins and were photographed using a digital camera from a fixed distance and with similar zoom (Fig. SII). The colored pins were inserted at different anatomical landmarks to help during digitalization of the landmark coordinates. A total of 11 landmark points were digitalized on each fish using the software TpsDig2^43^. For comparing the morphology of the specimens, we used the truss approach, which consists of the linear distances among morphological landmarks depicted upon the periphery of the objects^44^. Landmark coordinates were converted to linear distances using formula (1).

1 $$C=\sqrt{{a}^{2}+{b}^{2}}$$

where *a* and *b* denote the differences between the *x* and *y* coordinates of each pair of landmarks, respectively, and *C* is the linear distance between each pair of landmarks in pixels^44^. To compensate for size differences and allometric effects, all the distances were transformed to ratios related to the standard length of each specimen. The distance data were analysed with Principle Component Analysis (PCA) and Discriminant Function Analysis (DFA) in which the groups were defined before the analysis and the populations/groups were then analysed to find the most important discriminant variables. The discriminant functions were used to assign individuals to source groups. For DFA analysis, a forward stepwise approach was taken. To determine the relative importance of each morphological variable in discriminating different groups, *F*-to-remove statistics was set to 3.9 and other parameters left as default in SYSTAT9. In addition to the regular photos, x-ray photos of all specimens were taken to compare the position of the mouth opening.

### Mitochondrial *COI* analysis

DNA was extracted using the Chelex 100/200 method^45^ and the salt extraction method^46^. The primers FishF1 and FishR1 in Ward, *et al*.^19^ were used for *COI* amplification. The PCR reactions were performed in 25-µl volumes containing 18.5 µl H_2_O, 2.5 µl 10 × buffer, 0.5 µl MgCl_2_ (50 mM), 0.5 µl of each primer (10 mM), 0.5 µl dNTP, 0.5 µl Taq DNA polymerase, and 2 µl DNA solution. Thermal cycles included 1 cycle of 94 °C for 5 min; 35 cycles of 94 °C for 1 min, 58 °C for 1 min, and 72 °C for 1 min; and a final extension at 72 °C for 5 min. PCR products were sequenced using a forward primer and a Prisma 3130 sequencer following the protocols provided by the manufacturer (http://www.appliedbiosystems.com). The sequences were aligned and edited visually using Bioedit^47^.

### Nuclear DNA (nucDNA) analysis

#### Library preparation

DNA was extracted using salt extraction^46^ with an additional RNAse (Qiagen) treatment following the manufacturer’s protocol. The quality of the extracted DNA was checked on a 1% agarose gel and the degraded specimens were excluded. The extracted DNA was quantified using a NanoDrop spectrophotometer, and concentrations were normalized to 20 ng/µl (ranging from 16 ng/µl to 24 ng/µl) based on picogreen read values (Invitrogen: www.thermofisher.com). Libraries for Genotyping By Sequencing (GBS) were prepared following Mascher, *et al*.^48^. First, genomic DNA was digested using the *PstI* and *MspI* restriction enzymes, followed by ligation to a unique individual barcode and to adaptors for amplification. Barcoded specimens were multiplexed and amplified in a common tube. A total of 38 specimens were included per chip, for a total of three chips sequenced using the Ion Torrent technology available at the IBIS sequencing platform (Université Laval, Canada).

### Data processing and analysis

Sequencing adapters were removed using cutadapt^49^. Sequence quality of the first 10,000,000 reads was assessed using FastQC^50^. Libraries were de-multiplexed using process_radtags in STACKS V.1.35^27^. Reads were trimmed to 80 bp and shorter reads were discarded. We used the STACKS v1.35 analysis pipeline to score genotypes at 51,836 SNPs for our samples. Then these results were filtered using the 05_filter_vcf.py scripts included in stacks_workflow (https://github.com/enormandeau/stacks_workflow). The filtration parameters were: -l 0; -I 8; -p 70; -a 0.05; -A 0.05; -H 0.5; -f −0.5; -F 0.6; -s 10. A final set of 11,257 SNPs located on 7,048 reads were retained following the filtering steps.

The sequences of the 7,048 loci were concatenated and, finally, a 563,840-bp sequence per individual was produced for phylogenetic analyses. When heterozygous, the different SNPs were named using IUPAC symbols. On average, 6% of locus information was missing per individual, and missing alleles were imputed by the most frequent allele in each species for each locus.

To infer the historical demography based on Joint Allele Frequency Spectrum (JAFS) (see below), a dataset was prepared from the original VCF file. Several filtering steps – aimed at removing miscalled and low-quality SNPs, as well as false variation induced by merging paralogous loci – were performed using *VCFtools* v0.1.13^51^. SNPs with more than 90% of missing genotypes in all individuals were removed, but a lower exclusion threshold (50%) was applied to the out-group to retain a maximum of orthologous loci. After filtering for Hardy-Weinberg disequilibrium for each population (*p*-value exclusion threshold of 0.01), the filtered datasets were merged. Finally, the most parsimonious ancestral allelic state was determined by keeping monomorphic loci in the out-group, but polymorphic in the complex *G. typhlops*-*G. lorestanensis*, aiming to infer the divergence between species, of the studied complex. These result in 5,890 oriented SNPs used to build the unfolded JAFS.

### Detecting and characterizing hybridation

The admixture proportions among samples were inferred using the Bayesian clustering method implemented in the program STRUCTURE V.2.3.4^52^. The structure was evaluated for K = 1–5 using admixture model with correlated allele frequencies. The MCMC chains were ran for 100,0000 generations. The support for different values of *K* was assessed from the likelihood distribution (lowest cross-validation error) and visual inspection of the co-ancestry values for each individual. In addition, two supplementary *K-*means clustering analyses, the Bayesian Information Criterion (BIC; Schwarz^53^) and the Calinski–Harabasz pseudo-*F*-statistic^54^, were performed on individuals using the GENODIVE v.2.0b25 program^55^. For these *K*-mean clustering analyses, a simulated annealing method was used, where the optimal *K* value was determined via checking *K* values ranging from 1 to 5 for 5,000 permutations. A PCA implemented in the *ade4* package^56^ was performed and the first three principal components were visualized using *ggplot2* package available in R^57^.

### Phylogenetic analysis

The mtDNA and nucDNA phylogenetic trees were reconstructed using Neighbour-Joining (NJ) and Maximum Parsimony (MP) methods using MEGA7^58^ and Maximum Likelihood (ML) using RaxMLGIU 1.5b2^59^. The best-fit model(s) of mtDNA sequence evolution were selected using the online ModelTest^60^ in the HIV sequence database (http://hiv.lanl.gov/content/sequence/findmodel/findmodel.html). As there may be incongruence between the gene trees and species trees due to different factors like incomplete lineage sorting (ILS)^61,62^, species tree was reconstructed for the sequences of 5,843 loci, using SVDquartets + PAUP* (implemented in PAUP^*^4) software. SVDquartets inference of the species tree was performed using the multispecies coalescent tree model and QFM algorithm of quartet assembly. The branch supports in SVDqurtets method were calculated via implementing 1000 bootstrap repeats. *Garra mondica* was also included as an out-group. The K2P sequence distances for both data types were calculated using MEGA7. As the net molecular divergence (Da) has been reported to be a predictor of ongoing gene flow, this distance was also calculated^38^ using formula (2) (Camille Roux personal communication).

2 $$Da=({\rm{\Delta }}s-(\frac{{p}_{iA}+{p}_{iB}}{2}))/n$$

where ∆s is the average number of pairwise differences between sequences from species A and species B and *P*_*i*_ is the nucleotide diversity in each species, and *n* is the length of the concatenated sequence. The nucleotide differences and diversity indices used for the calculation of Da were calculated using MEGA7.

### Demographic history inferences

The demographic history of the species pair was inferred using *∂a∂i* v1.7^63^. The unfolded Joint Allele Frequency Spectrum (JAFS) was projected down to 5 individuals (*i.e*., 10 chromosomes), aiming to optimize the resolution and avoid remaining missing genotypes, which were not removed by the filtering threshold. Basic models of alternative modes of divergence: Strict Isolation (SI), Isolation-with-Migration (IM), Ancient Migration (AM), and Secondary Contact (SC) were tested. Briefly, each model consisted of the split of an ancestral population of effective population size *N*_ref_ in two populations of effective size *N*_*l*_ and *N*_*t*_ during a period of *T*_*split*_ (SI, IM) when populations diverged, *T*_*AM*_ + *T*_*S*_ (AM), or *T*_*S*_ + *T*_*SC*_ (SC) generations. The IM, AM and SC models allow migrant exchanges during *T*_*split*_, *T*_*AM*_ and *T*_*SC*_, respectively, at rate *m*_et->l_ from *G. typhlops* to *G. lorestanensis* and *m*_el->*t*_ in the opposite direction. We extended these models to integrate temporal effective population size variations (−*G*) aiming to describe expansions (*b*_*i*_ > 1) or bottlenecks (*b*_*i*_ < 1) in *b*_*l*_ and *b*_*t*_ for *G. lorestanensis* and *G. typhlops*, respectively, as implemented in Rougeux *et al*.^64^. The effective populations size variations started after the split of the ancestral population (SI and IM models) and after ancient migration and secondary contact for AM and SC models, respectively. In addition, models were extended to account for heterogeneous migration (−2*m*) across the genome. This parameter implementation allowed definition of two categories of loci. First, loci evolving neutrally (*i.e*., with migration rates *m*_et->l_ and *m*_el->*t*_) occurring in proportion *P* and the second for loci experiencing different effective migration rates (*i.e*., *m*_e′t->l_ and *m*_e′l->*t*_) due to their linkage with nearby genes under selection, occurring in proportion 1 − *P*^64,65^. The 14 tested models (Fig. SIII) were fitted independently applying successfully a hot and cold simulated annealing procedure followed by ‘BFGS’ optimization^65^. After running 25 independent optimizations for each model to obtain convergence, we retained the best one to perform comparisons among models based on Akaike information criterion (*AIC*). We defined a conservative threshold of ∆*AIC* = 10 and computed Akaike weights (*wAIC*) for models below the ∆*AIC* threshold^64^.

### Data availability

Demultiplexed DNA sequences are available at SRA database (SRA accession: SRP132073).NCBI Accession numbers for COI sequences: MG852030-MG852067.

## Electronic supplementary material

Supporting information
