# Supplementary material for: Genetic and morphological support for possible sympatric origin of fish from subterranean habitats
Source: Sci Rep. 2018 Feb 13;8:2909. doi: 10.1038/s41598-018-20666-w (PMC5811452; doi:10.1038/s41598-018-20666-w)
Supplement: Supplementary file 1 — Supporting information [file 41598_2018_20666_MOESM1_ESM.doc]

**Genetic and morphological support for possible sympatric origin of fish from subterranean habitats**

Iraj Hashemzadeh Segherloo^1*^, Éric Normandeau^2^, Laura Benestan^2^, Clément Rougeux^2^, Guillaume Coté^2^, Jean-Sébastien Moore^2^, NabiAllah Ghaedrahmati^3^, Asghar Abdoli^4^, Louis Bernatchez^2^

^1^ Department of Fisheries and Environmental Sciences, Faculty of Natural Resources and Earth Sciences, Shahr-e-Kord University, Shahr-e-Kord, P.B. 115, Iran^, 2^Département de biologie, Institut de Biologie Intégrative et des Systèmes (IBIS), Pavillon Charles-Eugène-Marchand 1030, Avenue de la Médecine Université Laval, Québec (Québec) G1V 0A6 Canada, ^3^ Lorestan Department of Environment, KhoramAbad, Iran, ^4^ Department of Biodiversity and Ecosystem Management, Environmental Sciences Research center, Shahid Beheshti University, Tehran, Iran

^*^email: [ihashem@nres.sku.ac.ir](mailto:ihashem@nres.sku.ac.ir)

Supporting information

Supplementary figure I. The JAFS projection from empirical (A) data and from the fittest model (B) and their residuals (C, D).


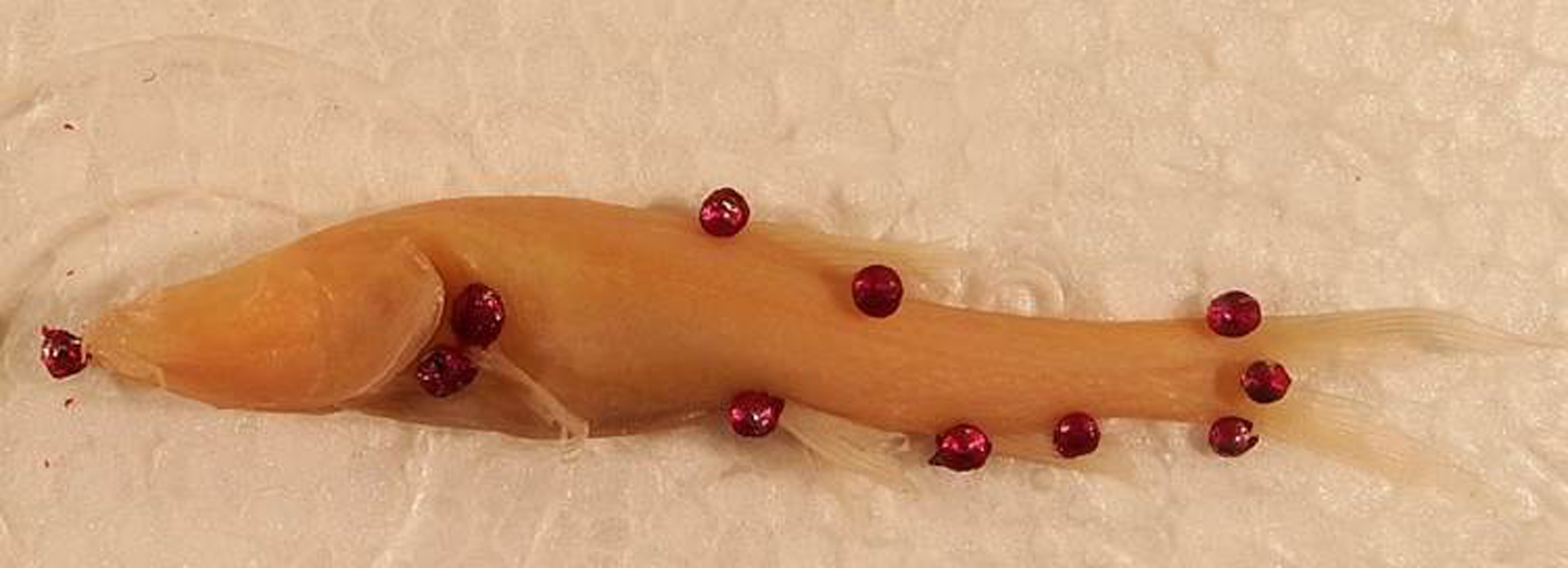


Supplementary figure II. Landmark points used for truss analysis of morphological analysis. The red colored pins denote 11 homologous landmark points around the fish specimens.

Supplementary figure III. The JAFS projection from empirical (A) data and form the fittest model (B) and their residuals (C, D).
